# Metachronous Testicular Germ-cell Tumors: The Importance of a Long-Term Follow-up

**DOI:** 10.4021/wjon2010.06.211w

**Published:** 2010-05-19

**Authors:** Filipe Nery, Diana Valadares, Franklim Marques

**Affiliations:** a Department of Internal Medicine, Centro Hospitalar do Porto - Hospital Santo Antonio, Portugal; b Director of Oncology Service, Centro Hospitalar do Porto – Hospital Santo Antonio, Portugal

**Keywords:** Metachronous, Testicular tumors, Bilateral

## Abstract

Testicular germ cell tumors (TGCT) are the most common malignancy in young male adults. They can be bilateral, and occur as a synchronous or metachronous tumor. The authors intend to characterize the prevalence and outcome of metachronous TGCT in the last 12 years of experience at our center. Cancer data base of our center was reviewed in order to find the patients that had TGCT in the period between 1996 and 2008 and, among those, the patients that had a second malignancy in the contralateral testicle after at least 6 months apart. Risk factors, clinical presentation, histological characteristics, staging, therapy and outcome were considered. Two out of 79 patients had metachronous TGCT, representing 2.5% of the group. Both cases had a low stage malignancy at the time of the diagnosis of the first tumor, and the diagnosis of the second TGCT happened 7 and 12 years later. Both patients are still alive without evidence of residual disease, under androgen replacement therapy and with testicular bilateral prostheses. Sperm cryopreservation was done in one of the patients. Long-term surveillance for TGCT is needed due to the probability of a second malignancy after the first 5 years of normal follow up. Special consideration must be given to patients submitted to bilateral orchiectomy concerning sperm cryopreservation, androgen replacement therapy and testicular prostheses.

## Introduction

Testicular cancer is an uncommon disease, representing 1 to 2% of all male cancers, in spite of being the most common malignancy among the youth [1, 2]. There are several risk factors for developing a testicular germ cell tumor (TGCT), such as infertility, cryptorchism, microcalcifications and genetic predisposition. Having had a previous TGCT is also a risk factor for developing a contralateral malignancy [3]. About 5% of the patients diagnosed with TGCT have already a contralateral testicular intraepithelial neoplasia (TIN) [4]. In various medical centers all over the world, the incidence of bilateral (synchronous and metachronous) TGCT varies between 1-7.8% [5-10].

The authors reviewed 12 years experience at Centro Hospitalar do Porto - Hospital Santo Antonio concerning bilateral metachronous TGCT, and tried to make a characterization of the risk factors, natural evolution and outcome of the disease.

## Case Report

Medical records of patients with testicular cancer between 1996 and 2008 were reviewed to identify all of those who developed a second TGCT. Eighty-five patients were found to have testicular cancer, but 79 were illegible as having TGCT. From those, we have only found 2 cases of bilateral metachronous TGCT described as follows.

### Case 1

An 18-year-old man, without any known risk factor for testicular malignancy, presented to our hospital with a painful right testicular mass with 1 month of evolution. Physical examination detected a small lump, confirmed by testicular ultrasound as a hypoechoic nodule. A CT-scan revealed no metastatic disease. α-fetoprotein (AFP) and lactate dehydrogenase (LDH) were above the normal limit. A right inguinal orchiectomy was performed and the histological exam revealed a mixed germ cell testicular tumor (composed by embrionary carcinoma and mature teratoma). Tumoral markers normalized after surgery, and the tumor was staged as pT1N0M0S0 - IA, according to the American Joint Committee on Cancer guidelines. The patient remained under surveillance. Twelve years later he developed bilateral gynecomastia and a high human chorionic gonadotropin (HCG). CT-scan found a 1cm lateroaortic adenopathy and a PET-scan revealed hyperfixation in the referred adenopathy and left testicle. At that time a scrotal ultrasonography was done, and a voluminous testicle of 5 x 5 x 3 cm with hypoechogenic and hypervascularized areas were found. This was followed by a left radical inguinal orchiectomy with testicle prosthesis introduction. Histological exam revealed a mixed TGCT (with seminoma, embrionary carcinoma and immature teratoma components) and a pathological stage pT2N1M0S1 – IIA. Chemotherapy was started with BEP protocol (bleomycin, etoposide and cisplatin), administered every 21 days for 3 cycles. Retroperitoneal adenopathy disappeared, HCG normalized and a surveillance program was initiated. The patient was disease-free in the 5 months after having finished his treatment.

### Case 2

A 29-year-old man developed a painless testicular lump, detected in auto-exam, and immediately sought medical assistance. An ultrasound examination showed a right testicle hypoechoic mass. AFP was above the superior limit of the normal value, and there was no evidence of metastatic disease in imagiological exams. Right inguinal radical orchiectomy was performed one day later, and histological exam revealed a teratoma with a low grade immature component. Pathological staging of pT1N0M0S0 – IA was done, and a surveillance program was implemented. Seven years later, during a routine scrotal ultrasound exam, a hypoechoic nodule of 11 mm was found. The patient was asymptomatic. Tumor markers were normal, as was the thoraco-abdomino-pelvian CT scan. A left inguinal radical orchiectomy was done. Histological exam revealed a TGCT with invasive classic seminoma with TIN component. He was staged as pT1N0M0S0 – IA, and a single carboplatin AUC7 session was done. 12 months later the patient remains disease free.

## Discussion

In spite of the comparatively low number of total patients (79) with TGCT in the last 12 years of experience, we have found 2 cases of metachronous TGCT, representing a prevalence rate of 2.5% as expected and described in the literature [5-10].

A diagnosis of metachronous testicular cancer is made when a gap of at least 6 months exists between the appearance of the first and second tumor, and when it is documented the inexistence of a contralateral mass by ultrasound examination performed at the time of the diagnosis of the first tumor [11]. Metachronous testicular tumors seems to be more frequent that the synchronous ones [6, 12].

A second testicular malignancy arises more often within 5 years after the first TGCT, in 70% of the cases [5]. In spite of that they can appear many years later, and metachronous testicular tumors have been reported 23 and 25 years after the first tumor and even up to 32 years after the first one [5, 13]

We report two cases where the second tumor appeared more than 5 years after the first one, 12 and 7 years respectively. This highlights the importance of long term surveillance when concerning TGCT follow-up, even in low stages of the first malignancy as we have described above. There is evidence of a 5-8% risk of TIN presence in contralateral testis that somehow can justify the need for long-term surveillance in order to diagnose the second TGCT as soon as possible. It seems this is so because within 7 years 70% of all TIN will progress to an invasive neoplasia [4, 14]. This risk is somewhat lower in patients that underwent chemotherapy for the first tumor. Biopsy of the contralateral testicle was not done to either patient at the time of surgery for the first tumor due to the fact that, in our institution, such procedure is done wherever testicule volume is lower than 12 ml.

Testicular autoexam and scrotal ultrasound examination are the main tools to recognize a second testicular cancer. However atypical presentation can arise as described for our first patient with gynecomastia being the only sign. HCG, also a tumoral marker, can be produced by testicular germ cell tumors, inducing breast overgrowth [15]. Their high blood levels, associated with gynecomastia, should lead the clinician to think about disease recidivation or a new TGCT.

Testicular tumors are, as already cited, more common in younger ages. It is always necessary to think about esthetic, sexual function and fertility. Testicular prostheses must be proposed to the patient, and they are usually well accepted [16]. Semen cryopreservation must be offered to the patient in the presence of a unique testicular cancer and when chemotherapy is planned, and to patients with metachronous or synchronous malignancies [17]. Due to the lack of testosterone production in patients submitted to bilateral orchidectomy, androgen replacement therapy should be started to avoid libido disorders, as well as important organic and psychological damage.

To both patients, testicular prostheses were implemented as well as androgen therapy started. Semen cryopreservation was done for the first patient, but not for the second one as he already had descendants.
